# Detection and Genetic Diversity of Bovine Kobuvirus in Diarrheic Calves in Türkiye: Genomic Characterization and Comparative B-Cell Epitope Analysis

**DOI:** 10.3390/ani16131973

**Published:** 2026-06-26

**Authors:** Selda Duran-Yelken, İlke Karayel-Hacıoğlu, Krisztián Bányai, Szilvia Marton, Feray Alkan

**Affiliations:** 1 Department of Virology, Faculty of Veterinary Medicine, Kastamonu University, Kastamonu 37150, Türkiye; syelken@kastamonu.edu.tr; 2 Department of Virology, Faculty of Veterinary Medicine, Ankara University, Ankara 06070, Türkiye; ikarayel@ankara.edu.tr; 3 Department of Virology, Graduate School of Health Sciences, Ankara University, Ankara 06070, Türkiye; 4 Department of Medical Biology, Medical School, University of Pécs, 7624 Pécs, Hungary; bkrota@hotmail.com; 5 Department of Pharmacology and Toxicology, University of Veterinary Medicine, 1078 Budapest, Hungary; 6 HUN-REN Veterinary Medical Research Institute, 1143 Budapest, Hungary; 7 HUN-REN-PE Limnoecology Research Group, 8200 Veszprém, Hungary; martonsil@gmail.com; 8 Research Group of Limnology, Center of Natural Sciences, University of Pannonia, 8200 Veszprém, Hungary

**Keywords:** Aichivirus B, bovine kobuvirus, B-cell epitope predictions, calf diarrhea, genomic characterization, Türkiye

## Abstract

Kobuviruses infect a wide range of hosts and exhibit high genetic diversity, highlighting the need for further studies to better understand their evolution and impact on animal health. In the present study, bovine kobuvirus (BKoV) was detected in 40 of 154 fecal samples from calves using the primers targeting the 3D gene region, corresponding to an overall positivity rate of 25.97%. All positive samples were obtained from diarrheic calves younger than 30 days of age, (40/133; 30.08%), consistent with previous studies. To enable more reliable genotyping, the complete VP1 gene sequences of seven BKoV strains were obtained, and the phylogenetic analyses showed that all VP1 sequences of this study clustered within Aichivirus B1. Additionally, immunoinformatics analysis of the VP1 protein identified conserved linear and discontinuous B-cell epitopes that may represent potential targets for cross-reactive diagnostic assays or future vaccine studies. The complete coding sequence of one strain, K056/2008/TUR, was also generated, and molecular analysis of this strain provided further evidence of ongoing viral evolution and genetic diversity in Türkiye. Overall, these findings provide new insights into the prevalence, genetic diversity, and predicted immunogenic features of BKoVs in calves in Türkiye, supporting further studies on its pathogenesis, diagnostics, and antigenic properties.

## Simple Summary

Bovine kobuvirus (BKoV) has been detected in fecal samples from cattle with and without diarrhea in various countries. However, information on its prevalence, genetic diversity, and antigenic characteristics remains limited. In this study, fecal samples from calves (n = 154) were screened for BKoV and viral RNA was detected in only diarrheic calves younger than 30 days of age (40/133; 30.08%). Complete VP1 gene sequences of seven strains and the complete coding sequence of one strain were analyzed to better understand the genetic characteristics of BKoV circulating in Türkiye. In addition, predicted B-cell epitopes in the VP1 protein were identified using immunoinformatics approaches. The results showed that Turkish BKoV strains clustered within Aichivirus B1 and contained conserved epitope regions that may be useful for future diagnostic or vaccine-related studies. These findings contribute to a better understanding of BKoV diversity and its potential relevance in calf diarrhea.

## 1. Introduction

Calf diarrhea is a leading cause of morbidity and mortality in calves worldwide, resulting in substantial economic losses for the cattle industry. Various infectious agents, including viruses, bacteria, and protozoa, can cause calf diarrhea [1]. In addition to the main viral diarrheal agents such as rotavirus A and bovine coronavirus (BCoV), various agents such as bovine kobuvirus (BKoV), norovirus, and nebovirus have also been detected in diarrheic calves [1,2,3]. To date, kobuviruses (KoVs) have been identified in humans as well as in a wide range of domestic and wild animals, with or without clinical symptoms [4,5,6]. However, despite increasing reports of detection, the clinical significance and pathogenic role of several of these viruses, particularly KoVs, remain poorly characterized.

The genus *Kobuvirus*, belonging to the family *Picornaviridae*, is currently classified into six species: *Kobuvirus aichi*, *Kobuvirus bejaponia*, *Kobuvirus cebes*, *Kobuvirus dekago*, *Kobuvirus ecuni*, *and Kobuvirus femyomini*, which were previously known as Aichivirus A to F, respectively. *Kobuvirus bejaponia*, formerly known as Aichivirus B, includes KoVs detected in cattle (Aichivirus B1, commonly referred to as BKoV), ferrets (Aichivirus B2), and sheep (Aichivirus B3) [7]. BKoV is a non-enveloped, single-stranded, positive-sense RNA virus with a genome of approximately 8.3 kb. The genome has 5′ and 3′ untranslated regions (UTRs) and a single open reading frame (ORF) encoding a polyprotein that is processed into structural (P1) and non-structural proteins (P2 and P3) [8]. The genomic organization is as follows: 5′-L-VP0-VP3-VP1-2A-2B-2C-3A-3B-3C-3D-3′. Because of its high variability, VP1 is an excellent candidate for genotyping as an immunological determinant protein [3,9,10].

Since the first identification of Aichivirus in humans in Japan [11] and the subsequent description of BKoV in cattle [12], members of the genus *Kobuvirus* have attracted increasing scientific attention worldwide. Over decades, KoVs have been detected in a broad range of animal species, including cattle, pigs, sheep, goats, dogs, cats, rodents, and wildlife, indicating their extensive host diversity and global distribution [13,14,15,16]. Parallel advances in molecular diagnostics and sequencing technologies have facilitated numerous epidemiological and genetic characterization studies, substantially improving our understanding of KoV evolution, genomic diversity, and host adaptation [13,17]. In particular, genetic analyses based on structural and non-structural genomic regions have revealed considerable heterogeneity among kobuvirus strains circulating in different geographic regions and host species. These developments have highlighted the importance of continuous molecular surveillance and comprehensive genomic characterization of KoVs to better understand their epidemiology and evolutionary dynamics [12,18,19,20,21,22,23,24]. Therefore, this study aimed to characterize BKoV strains circulating in Türkiye by combining full genome sequencing with detailed analysis of the VP1 region, including molecular characterization and in silico prediction of B-cell epitopes.

## 2. Materials and Methods

### 2.1. Diagnostic Samples

A total of 154 fecal samples from calves under six months of age were used in this study. The animals were recorded in broad age categories (<30 days, <3 months, and <6 months) and included calves with diarrhea (n = 133) and without diarrhea (n = 21). Samples were collected from 56 cattle farms, with 53 of them providing specimens from diarrheic animals, across 11 provinces (Aksaray, Amasya, Ankara, Bursa, Çankırı, Eskişehir, İzmir, Kars, Kırklareli, Konya, and Şanlıurfa) between 2008 and 2024. Fecal samples were stored at −80 °C until RNA extraction.

### 2.2. Nucleic Acid Extraction, RT-PCR for the Detection and Genotyping of BKoV

The viral RNA was extracted from fecal sample suspensions at a dilution ratio of 1:10 (weight/volume) using the GENEzol™ TriRNA Pure Kit (Geneaid, New Taipei City, Taiwan) following the manufacturer’s instructions and was stored at −80 °C. The cDNA was synthesized using the RevertAid First Strand cDNA Synthesis Kit (Thermo Fisher Scientific, Waltham, MA, USA), according to the manufacturer’s instructions.

Initially, BKoV were screened by RT-PCR using primers targeting the 3D gene region (encoding the RNA-dependent RNA polymerase, RdRp) [22]. Amplification was performed using DreamTaq DNA Polymerase (Thermo Fisher Scientific, Waltham, MA, USA), and the optimized PCR thermal conditions were performed as follows: denaturation at 95 °C for 3 min, 35 cycles of denaturation at 95 °C for 30 s, annealing at 55 °C for 30 s, extension at 72 °C for 1 min, and final extension at 72 °C for 10 min. Subsequently, the samples yielding expected size (1032 bp) amplicons were subjected to further RT-PCR using the primers targeting the BKoV VP1 gene region (890 bp) [22]. The cycling conditions were as follows: initial denaturation at 95 °C for 15 s, 35 cycles of denaturation at 95 °C for 1 min, annealing at 50 °C for 1 min, extension at 72 °C for 1 min, and a final extension at 72 °C for 10 min. For each RT-PCR assay, a previously confirmed BKoV-positive sample as a positive control and nuclease-free water as a no-template negative control were used. PCR products were electrophoresed on a 1% agarose gel stained with SafeView™ Classic (ABM, Richmond, BC, Canada) and visualized under UV light. All amplified VP1 fragments were submitted to a commercial sequencing service and sequenced in both directions using the same primers as those employed for RT-PCR.

### 2.3. Genome Sequencing and Bioinformatic Analysis

Fragments of the genome sequence of a BKoV (K056/2008/TUR) strain, whose VP1 gene was successfully amplified in this study, were retrieved by reanalyzing data from a previous whole-genome sequencing study of rotavirus A strains conducted on the same sample [25]. Whole-genome sequencing of the virus strain was carried out following established protocols [26,27]. Briefly, after nuclease treatment, viral RNA was extracted using the Direct-zol RNA MiniPrep Kit (Zymo Research Corporation, Irvine, CA, USA) according to the manufacturer’s instructions. Subsequently, random-primed RT-PCR was performed to generate DNA for high-throughput sequencing. A total of 10–100 ng template DNA obtained by random PCR was subjected to enzymatic fragmentation and adaptor ligation using the NEBNext fast DNA fragmentation and library prep set for Ion Torrent Kit (New England Biolabs, Ipswich, MA, USA) according to the manufacturer’s instructions. The adaptor ligation was performed using reagents from the same kit, while the barcoded adaptors were obtained from the Ion Xpress barcode adapters kit (Life Technologies Inc., Waltham, MA, USA). The barcoded library DNA samples were run on a 2% precast gel after column-based extraction, and products of approximately 300–350 bp were used in PCR amplification using the NEBNext fast DNA fragmentation and library prep set for Ion Torrent kit (New England Biolabs, Ipswich, MA, USA). Library DNA was amplified under the following conditions: initial denaturation at 98 °C for 30 s; 12 cycles of 98 °C for 10 s, 58 °C for 30 s, and 65 °C for 30 s; and final extension at 65 °C for 5 min. The amplified library DNA was purified using a gel/PCR DNA fragments extraction kit (Geneaid, New Taipei City, Taiwan) and quantified on a Qubit 2.0 fluorometer using the Qubit dsDNA BR Assay Kit (Invitrogen, Carlsbad, CA, USA). Emulsion PCR for clonal amplification of the libraries was performed according to the manufacturer’s protocol using the Ion PGM Template OT2 200 Kit with Ion OneTouch v2 equipment. Enrichment of the templated beads (on an Ion OneTouch ES system) and further steps of pre-sequencing set-up were carried out according to the 200 bp sequencing protocol recommended for 316 chips. Raw sequencing data were analyzed using CLC Genomics Workbench version 7 (CLC Bio-Qiagen, Aarhus, Denmark). By using a combination of de novo assembly and reference sequence mapping, an initial BKoV-related consensus sequence was obtained for the BKoV strain K056/2008/TUR. To complete the remaining sequence gaps, supplementary RT-PCR analyses were conducted using the primer sets listed in Table S1. The resulting gap-filling PCR products were subjected to Sanger sequencing, and the obtained sequences were incorporated into the final consensus sequence.

### 2.4. Sequence Analysis and Phylogenetic Methods

The generated sequence data were analyzed using the BLASTn service provided by the National Center for Biotechnology Information (NCBI). Sequences were aligned using AliView [28] and the MUSCLE algorithm [29]. Multiple sequence alignments were conducted with all available KoV sequences obtained from GenBank. The optimal substitution model for Maximum Likelihood (ML) phylogenetic analysis was determined using the “Find Best DNA/Protein Models” tool in MEGA X [30]. The nucleotide substitution model was selected according to the Bayesian Information Criterion (BIC). The ML phylogenetic trees were constructed using the GTR+G+I nucleotide substitution model for the complete coding sequence (CDS) encoding the polyprotein, P1, P2, and P3 regions, and the GTR+G model for VP1. The nucleotide (nt) and amino acid (aa) identities were calculated using the SIAS online tool (http://imed.med.ucm.es/Tools/sias.html (accessed on 19 February 2026)). The sequences generated in this study were deposited in GenBank under the accession numbers PZ284860-PZ284866.

### 2.5. Recombination Analysis

Potential recombination events in the near-complete genome sequence of the K056/2008/TUR strain were investigated using the Recombination Detection Program version 4.100 (RDP4) [31]. A representative dataset was generated based on the phylogenetic trees shown in Figure 2a–d in the Results section. Recombination signals were screened using the RDP, GENECONV, BootScan, MaxChi, Chimaera, SiScan, and 3Seq methods implemented in RDP4, with the highest acceptable *p*-value set to 0.05 and Bonferroni correction applied. Events supported by at least four out of the seven methods were considered for interpretation. Potential recombination signals identified by RDP4 were further evaluated using similarity plot analysis in SimPlot software version 3.5.1. SimPlot analysis was performed using a window size of 200 nt and a step size of 20 nt.

### 2.6. B-Cell Epitope Prediction

Three-dimensional structural models of the VP1 protein of BKoVs were predicted using AlphaFold via the ColabFold pipeline (AlphaFold2.ipynb) [32]. Amino acid sequences were used as input, and predicted structures were obtained in PDB format for subsequent discontinuous (conformational) B-cell epitope analysis.

B-cell epitope prediction was conducted by analyzing the amino acid sequences of the BKoV VP1 capsid gene using the online B-Cell Epitope Prediction Tool: The Immune Epitope Database (IEDB), accessible at www.iedb.org (accessed on 23 June 2026) [33]. This tool was utilized to identify both linear (continuous) B-cell epitopes and discontinuous B-cell epitopes (3D structure of the antigen) derived from the primary amino acid sequence. Bepipred Linear Epitope Prediction 2.0 and Kolaskar & Tongaonkar antigenicity method were employed for predicting continuous B-cell epitopes [34,35]. For BepiPred 2.0, the default threshold value of 0.5 was used, and residues with scores above this threshold were considered predicted epitope residues. For Kolaskar & Tongaonkar method, the sequence-specific threshold calculated automatically by the server was used for each VP1 sequence. Regions showing positional overlap between BepiPred 2.0 and Kolaskar & Tongaonkar predictions were considered overlapping predicted linear B-cell epitope regions. To predict discontinuous B-cell epitopes, the online ElliPro tool was used. In this approach, the minimum score and maximum distance (in Å) were set to 0.5 and 6, respectively [33,36].

### 2.7. Statistical Analysis

The association between BKoV detection and diarrheic status was evaluated using Fisher’s exact test. Statistical analyses were performed using IBM SPSS Statistics for Windows, version 27.0 (IBM Corp., Armonk, NY, USA). A *p*-value < 0.05 was considered statistically significant. The risk difference and its 95% confidence interval were calculated using the score method in R software 4.6.0.

## 3. Results

### 3.1. Detection of BKoVs and Analysis of Their VP1 Gene Region

The results of the RT-PCR, which was performed to detect the presence of the 3D gene fragment, revealed that 40 out of 154 samples (25.97%) from calves exhibited positive outcomes. The positivity rates were 35.7% (20/56) among all sampled farms and 37.7% (20/53) among farms with cases of diarrhea. All BKoV-positive samples were obtained from diarrheic calves (40/133; 30.08%), whereas none of the non-diarrheic calves tested positive (0/21; 0%). Fisher’s exact test indicated a statistically significant association between BKoV detection and diarrheic status (*p* = 0.002). The risk difference between diarrheic and non-diarrheic calves was 30.1% (95% CI: 14.0%–38.4%). Additionally, all BKoV-positive calves were younger than 30 days of age.

**Figure 1:**
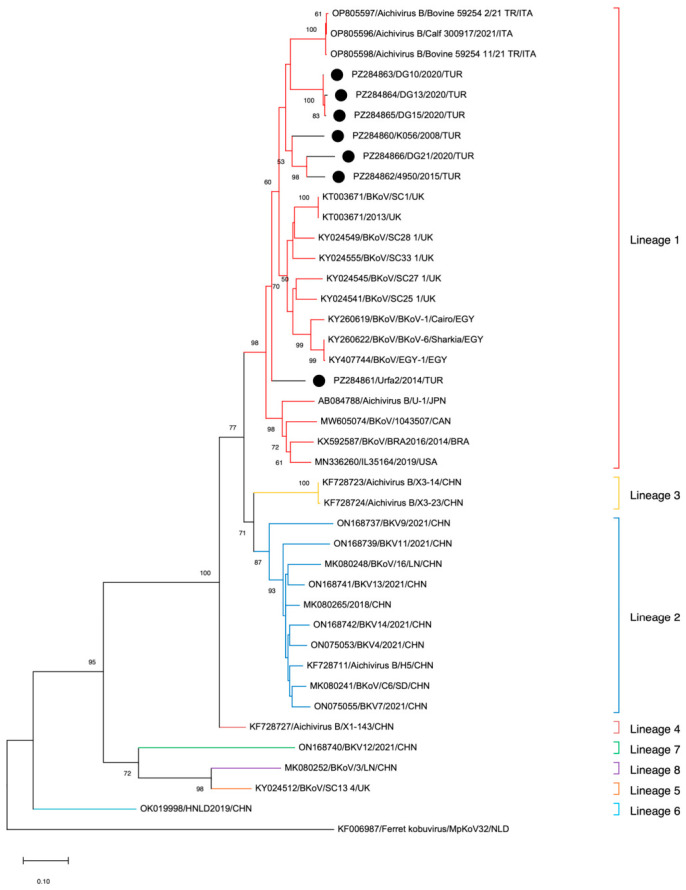
Phylogenetic tree based on the nucleotide sequences of the VP1 gene (801 bp) of BKoV strains. The ML phylogenetic tree was constructed with the GTR+G nucleotide substitution model with 1000 bootstrap replicates. The study strains are indicated with black dots. The tree was rooted using Ferret kobuvirus/MpKoV32/NLD (KF006987) as an outgroup, and bootstrap values ≥50% are present. The scale bar indicates nucleotide substitutions per site.

In this study, the VP1-encoding gene was successfully amplified and sequenced from seven samples (K056/2008/TUR, Urfa2/2014/TUR, 4950/2015/TUR, DG10/2020/TUR, DG13/2020/TUR, DG15/2020/TUR, and DG21/2020/TUR), which were collected from calves across six different farms. DG10/2020/TUR and DG21/2020/TUR originated from the same farm in 2020, whereas all other samples were obtained from different farms and collected between 2008 and 2020. VP1 gene sequence analysis revealed that all strains identified in this study belonged to Aichivirus B1 (Figure 1), and shared 84.64–99.37% nt and 91.38–99.62% aa identity among themselves. The phylogenetic tree based on the VP1 gene—known for its high variability and located within the P1 region—demonstrated that all strains identified in this study clustered within Lineage 1, according to the classification proposed by Li et al. [37] which defines eight distinct lineages. Lineage 1 contains BKoV strains reported from the United Kingdom, Egypt, Brazil, Japan, Canada, the USA, Italy, as well as the strains identified in this study from Türkiye (Figure 1). In detail, three strains from this study, DG10/2020/TUR, DG13/2020/TUR, and DG15/2020/TUR, clustered tightly together, forming a distinct subcluster. In contrast, the other three strains (K056/2008/TUR, 4950/2015/TUR, and DG21/2020/TUR) were assigned to a distinct branch, whereas Urfa2/2014/TUR was positioned on a separate and independent branch, indicating further phylogenetic divergence.

### 3.2. Genome Sequence Analyses of the BKoV Strain, K056/2008/TUR

In this study, the genome sequence of a BKoV strain from Türkiye, K056/2008/TUR (PZ284860), was determined. However, approximately 350 nt at the 5′ end of the 5′UTR are missing; therefore, the sequence represents a near-complete genome. The genome is 7950 nt in length and comprises a 420 nt 5′UTR, a 7392 nt annotated coding sequence (CDS), and a 138 nt 3′UTR. The CDS represents the complete protein-coding region of the genome and encodes a 2463 amino acid polyprotein, starting with a methionine codon and terminating with an alanine codon (Table 1).

**Figure 2:**
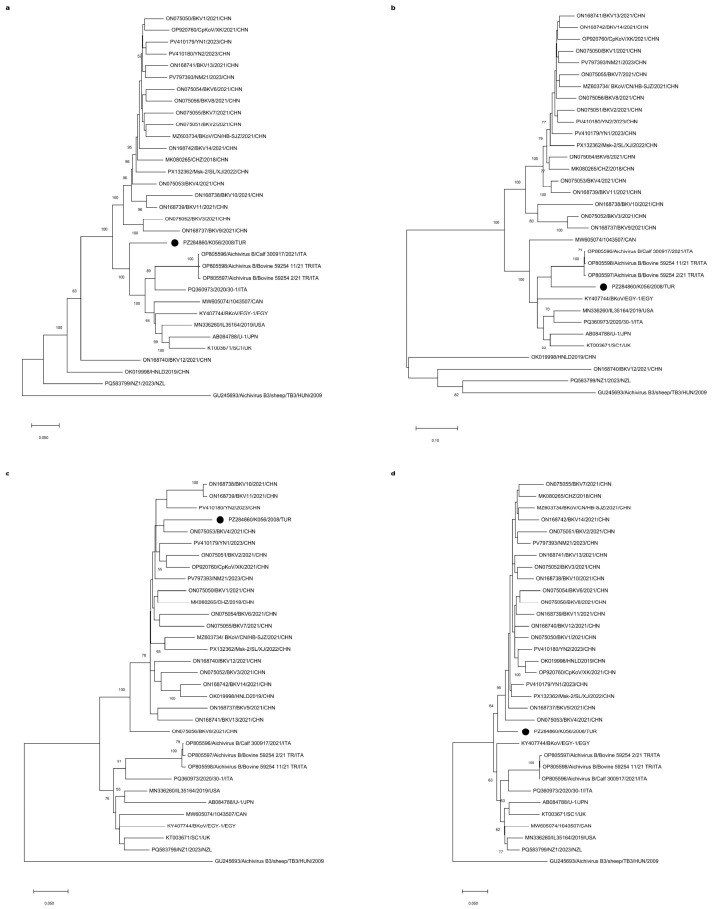
The phylogenetic trees, based on the nucleotide sequences of the complete CDS (**a**), P1 (**b**), P2 (**c**), and P3 (**d**) regions of BKoV strain K056/2008/TUR and BKoV strains available in GenBank. The ML phylogenetic trees were constructed using the GTR+G+I nucleotide substitution model with 1000 bootstrap replicates. The strain K056/2008/TUR is indicated with a black dot. The trees were rooted using AichivirusB3/sheep/TB3/HUN/2009 (GU245883) as an outgroup and bootstrap values ≥50% are present. The scale bar indicates nucleotide substitutions per site.

The genomic sequence exhibited a G + C content of 55.5%. Furthermore, compared with other Aichivirus B strains from ruminants with complete CDS, the genome of the strain K056 shared 81.43–90.27% nt identity and 86.21–97.25% aa identity (Tables S2 and S3). The 2C region of the strain K056/2008/TUR is conserved with 85.87–93.73% nt and 93.13–100% aa identity and includes the highly conserved motif GPPGTGKS, which is the nucleotide-binding domain of the putative picornavirus helicase. The three highly conserved motifs, KDELR, YGDD, and FLKR, in the 3D region encoding RdRp were also identified, and the nt and aa identities of this region were found to be 90.7–95.95% and 95.74–99.14%, respectively. The nt and aa identities at the individual gene and P1–P2–P3 regions levels for the strain K056/2008/TUR to other Aichivirus B strains of different countries were shown in Tables S2 and S3. The phylogenetic analysis, based on the nucleotide sequences of the complete CDS, P1, P2, and P3 regions of the strain K056/2008/TUR, is presented in Figure 2a–d.

### 3.3. Results of the Recombination Analysis

Recombination analysis of the representative dataset identified a putative recombination evidence in the near-complete genome sequence of the K056/2008/TUR strain. The RDP4 program reported that it was detected between nucleotide positions 3166 and 6093, and the beginning breakpoint could not be precisely resolved. In this event, Italy-origin (OP805597) and China-origin (ON168738) BKoV strains were inferred as the putative major and minor parental sequences, respectively. The event was supported by six recombination detection methods, including RDP, BootScan, MaxChi, Chimaera, and SiScan. Similarity plot analysis supported the RDP4 result by showing a shift in sequence similarity across the predicted recombinant region (Figure S1).

### 3.4. Prediction of Linear and Discontinuous B-Cell Epitope

Comparative analysis of linear B-cell epitope predictions, based on the combined results of BepiPred 2.0 and Kolaskar & Tongaonkar antigenicity methods, revealed three predicted major epitope regions within the VP1 protein (Table S4). All three regions (~64–73, ~140–149, and ~248–256/257) were detected in all seven BKoV strains analyzed (Table 2). Among these, the ~248–256/257 region showed the highest level of conservation and confidence, being consistently predicted across all seven strains and supported by both sequence-based methods. The ~64–73 region was also conserved across all strains but frequently exhibited shorter overlapping amino acids, while the ~140–149 region showed more variable and often fragmented overlaps among strains (Table 2).

**Figure 3:**
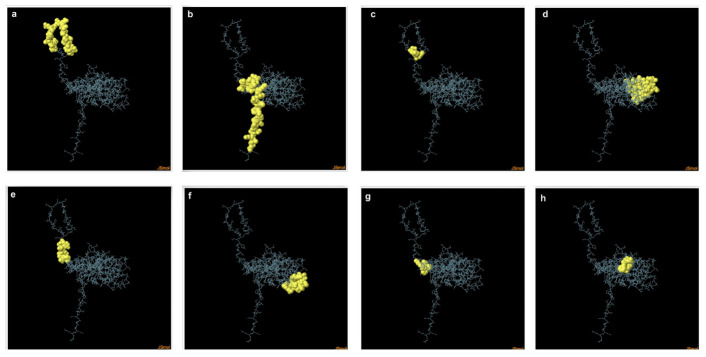
Visualization of predicted discontinuous B-cell epitopes on the three-dimensional structure of the VP1 capsid protein of the strain K056/2008/TUR using ElliPro. Images (**a**–**h**) represent the spatial localization of residue clusters corresponding to epitope regions in No. 1–8, respectively, as listed in Table S5. Residues forming each predicted discontinuous epitope are highlighted in yellow.

Discontinuous B-cell epitope analysis of VP1 protein models from our seven BKoV strains was performed using ElliPro to evaluate whether the conserved linear B-cell epitope regions identified by sequence-based analyses were predicted to be exposed on the protein surface (Table S5). The predicted discontinuous B-cell epitopes of the VP1 capsid protein of the strain K056/2008/TUR using the ElliPro server are shown in Figure 3. Across all VP1 models, ElliPro predicted between 8 and 10 conformational epitope clusters per isolate, comprising a total of 124–135 amino acid positions, with epitope scores ranging from 0.509 to 0.936 (Table S5). Consistent with the linear epitope analysis, the three conserved epitope regions (~64–73, ~140–149, and ~248–256/257) were further evaluated in a structural context to assess their surface-exposed likelihood and potential overlap with discontinuous B-cell epitopes predicted by ElliPro tool. All three regions were represented within ElliPro-predicted conformational epitopes in all seven isolates, suggesting that these conserved sequence-based epitope regions may be structurally exposed and potentially accessible on the VP1 surface. Among these, the ~248–256/257 region was consistently encompassed by high-scoring ElliPro epitopes (scores generally >0.80), suggesting a higher predicted degree of structural protrusion and possible accessibility on the VP1 surface. In contrast, the ~64–73 and ~140–149 regions exhibited partial or fragmented overlap with predicted conformational epitopes across the seven strains. In addition to these conserved linear regions, ElliPro predicted several high-scoring conformational epitope clusters that were frequently located in the N-terminal region of the VP1 protein. Across strains, a conserved predicted cluster spanning residues 1–25 was consistently identified, with ElliPro scores generally exceeding 0.80 (Table S5), suggesting substantial predicted structural protrusion and potential surface exposure (Figure 3). The epitopes numbered 1–8 in Table S5 correspond to panels a–h in Figure 3, respectively.

## 4. Discussion

It is well established that enteric diseases continue to represent a significant challenge for the livestock industry. A multitude of viruses have been identified as the etiological agents of diarrhea in livestock, including the well-known rotavirus A and BCoV [1,2,3]. In addition, there has been a notable increase in the incidence of novel viruses such as kobuvirus, hunnivirus, and bopivirus included in the *Picornaviridae* [9,20,38]. Following the initial identification, BKoV has been detected in numerous countries with the prevalence rates between 1 and 77.8%, indicating global distribution in cattle exhibiting either diarrhea or non-diarrhea [9,10,12,20,21,22,39]. In this study, a total of 154 samples were analyzed, of which 40 (25.97%) tested positive. All positive samples were obtained from the diarrheic calves (40/133; 30.08%), whereas no positivity was detected among non-diarrheic calves (0/21; 0%) (Fisher’s exact test, p = 0.002). Although this result supports an association between BKoV detection and diarrhea, it should be interpreted cautiously because of the relatively small non-diarrheic group. A previous study conducted in Türkiye [20], representing the only available prevalence data on BKoV in the country, reported a detection rate of 22.8% in calves with diarrhea, which is comparatively lower than the rate observed in the present study. Furthermore, studies from various countries have reported widely varying BKoV detection rates in diarrheic calves, ranging from 5.2% to 66.7% [9,19,22,23,40]. In addition, the detection rate of BKoV in fecal samples from diarrheic calves was higher than in non-diarrheic calves in several reports [19,37,41], supporting its potential association with diarrhea, which is consistent with the findings of the present study. Also, Wang et al. [23] reported that the results of histopathological examination of calves with diarrhea clearly demonstrated viral infection, with only BKoV being detected, providing evidence that BKoV may play a role in calf diarrhea. Further research, including virus isolation and virus challenge to calves, is required to elucidate the role of BKoV infection in cattle. In regard to the age of infected calves, the findings of this study demonstrated a notable common occurrence in calves younger than 30 days. Although the frequent detection of infection in calves younger than 30 days of age is consistent with previous studies [21,22,41], it should be interpreted with caution due to the cross-sectional nature of the study and the inclusion of different age and clinical groups. The limited and uneven distribution of samples across age groups, together with the small number of animals in certain categories, also precluded meaningful statistical comparisons. Therefore, the relationship between infection and age should be considered a descriptive observation rather than evidence of a distinct age-associated risk pattern. Also, given the high prevalence of coinfections in diarrheic calves, particularly during the first month of postnatal life, the need for a more cautious interpretation of the relationship between age and infections evident. Therefore, data on additional viral pathogens such as rotavirus A, BCoV, and bovine caliciviruses (BNoV and BNeV) derived from previous studies [25,42,43] and routine diagnostic analyses were evaluated for BKoV-positive samples, and several coinfection findings were identified (Table S6). The data indicated that none of the investigated viruses was detected in 67.5% (27/40) of the BKoV-positive animals and in 35% (7/20) of the farms. However, although mentioned viruses are considered major etiological agents of calf diarrhea, the involvement of other viral, bacterial, and parasitic pathogens certainly cannot be completely ruled out. Briefly, several coinfection findings detected among BKoV-positive calves and/or their sampled farms suggest that BKoV may contribute to calf diarrhea both as part of coinfections and, in some cases, as a possible sole etiological agent, supporting its potential involvement in diarrheic calves.

In contrast to the 3D gene region, which is relatively more conserved within the BKoV genome, the VP1 gene has been suggested to be more suitable for genotyping due to its higher genetic variability [3]. Therefore, this study employed complete sequencing of the VP1-encoding gene, enabling a more reliable determination of BKoV genotypes, representing, to the best of our knowledge, the first report of complete VP1 gene sequencing for BKoVs in Türkiye. Although 40 fecal samples, obtained between 2008 and 2024 (Table S6), were positive for BKoV by screening PCR, the VP1 amplicons were obtained from only seven samples (17.5%). Amplification failure in the remaining positives was most likely associated with low viral RNA levels, RNA degradation, the presence of fecal PCR inhibitors, and/or sequence variability in primer-binding regions. Phylogenetic analysis showed that all seven BKoV strains, comprising one strain each from 2008, 2014, and 2015, and four strains from 2020, clustered within the Lineage 1 (Figure 1), the most prevalent cluster worldwide. The observed formation of multiple subclades within this lineage may also suggest genetic diversity in the VP1 region, which could contribute to antigenic variability among the analyzed BKoV strains. The inclusion of sequences from the temporally distant period spanning 2008 to 2020 provides a broader temporal context, despite the limited number of sequences and the absence of VP1 data from more recent samples. Nevertheless, further studies with expanded sampling are needed to determine the extent and persistence of this lineage in Türkiye.

As previously stated, among the three capsid proteins, VP1 is the most surface-exposed and structurally variable protein on the viral particle [3,12]. Zhu et al. [44] suggested that VP1 is involved in enteric receptor recognition and may contribute to viral pathogenesis [44]. Moreover, this protein is considered an immunodominant and is responsible for eliciting neutralizing antibodies [3,45]. B-cell epitopes, also known as antigenic determinants, are specific clusters of amino acids on an antigen that are recognized by secreted antibodies or B-cell receptor [46]. The presence of multiple subclades within lineage 1 (Figure 1) suggests ongoing genetic diversification in the VP1 region of BoKVs, which may contribute to antigenic variability. Due to the phylogenetic positions of our strains as defined in Figure 1, a comparative immunoinformatic analysis was performed to evaluate both linear and discontinuous B-cell epitopes within their VP1 proteins. It should be emphasized that these analyses were based solely on computational predictions, and therefore the predicted epitopes should be interpreted as regions of preliminary interest rather than experimentally validated antigenic determinants. This analysis identified three predicted conserved regions (~64–73, ~140–149, and ~248–256/257) among all strains, whereas other predicted epitope regions displayed one or several amino acid substitutions, reflecting potential antigenic variation (Table 2 and Table S4). ElliPro-based structural analysis further predicted that these conserved sequence-based regions may correspond to conformational epitopes with varying degrees of surface exposure. Notably, the ~248–256/257 region exhibited the highest level of sequence conservation and the most consistent predicted structural protrusion across VP1 models, suggesting that it may represent a potential region for further experimental evaluation of cross-strain antibody recognition. In addition, ElliPro consistently identified a high-scoring conformational epitope cluster within the N-terminal segment of VP1 (residues 1–25) in all strains. Although this region was not detected by linear epitope prediction methods, its identification in structure-based analysis suggests that the VP1 N-terminus may also represent a potentially accessible conformational region, pending experimental confirmation. A similar pattern of conformational VP1 epitopes has been reported for canine kobuvirus in which discontinuous epitope clusters were detected in the N-terminal region (residues 1–32) as well as within the 234–274 region of VP1 [13]. Although these predictions provide a useful preliminary framework, additional evaluations including epitope conservancy, surface accessibility, antigenicity, immunogenicity, cross-reactivity, and other related parameters, etc., are necessary to determine their suitability for diagnostic assay development or other biological applications. Therefore, experimental validation using peptide-based assays, serological approaches, virus neutralization assays, or monoclonal antibody mapping is required to determine their functional relevance. Future studies incorporating these analyses will be necessary to clarify the biological significance of the predicted epitopes.

In this study, we also report, for the first time, the complete CDS of the Turkish BKoV strain, K056/2008/TUR. Pairwise sequence comparison of the CDS revealed that K056/2008/TUR shared the highest identity with the Chinese BKoV4/2021/CHN strain (ON075053) among the BKoV strains from different countries, with 90.27% nt and 97.2% aa identity. The 10% difference in nucleotide sequence between the K056/2008/TUR strain and the most closely related strain lends support to the hypothesis put forth in a previous study [21] that the virus can persist and evolve in the field for extended periods. Moreover, the phylogenetic tree based on the complete CDS showed that strain K056/2008/TUR clustered within the Aichivirus B1 lineage and formed an independent branch. In detail, K056/2008/TUR was positioned as a neighboring branch to a broader subgroup comprising Italy-, Canada-, Egypt-, USA-, Japan-, and UK-origin strains (Figure 2a). The phylogenetic tree based on the P1 region was largely consistent with the CDS-based tree; however, in this analysis, K056/2008/TUR showed a closer relationship with the Italy-origin strains (88.0–88.1% nt identity) (Figure 2b). In contrast, the P2 region-based tree placed K056/2008/TUR within a broader cluster of Chinese BKoV strains (Figure 2c). The P3-based phylogenetic tree positioned K056/2008/TUR on a neighboring branch outside the main Chinese cluster, rather than directly clustering it with either the Chinese or Italy-origin subgroups (Figure 2d). These findings suggest that K056/2008/TUR represents a relatively distinct variant within the currently available complete-genome BKoV sequences. In line with this observation, RDP4 and similarity plot (Figure S1) analyses supported a putative recombination signal in K056/2008/TUR. Therefore, the distinct placement of K056/2008/TUR in the P1, P2, and P3 trees may be at least partly associated with a possible recombination event. The beginning breakpoint could not be precisely resolved; therefore, this finding should be interpreted cautiously as a putative recombination event. Moreover, given the limited number of complete or near-complete BKoV genomes currently available, it should not be overlooked that the inferred parental strains may represent the closest available relatives rather than the definitive parental viruses. Nevertheless, this finding suggests that recombination may contribute to BKoV genomic diversity and highlights the need for additional complete-genome data to clarify the evolutionary origin and significance of K056/2008/TUR. To date, limited complete genome sequences of BKoV have been deposited in GenBank. These sequences were derived from Japan [12], the UK (unpublished), Egypt [22], China [18,37,39], the USA [23], Canada [21], Italy [10,47], and New Zealand [48]. Considering the increasing detection of BKoV in association with diarrheal disease in cattle, further whole-genome sequencing studies are essential to better understand its genetic diversity and evolutionary dynamics, and potential clinical relevance.

The present study has several limitations that should be considered when interpreting the findings. Briefly, the limited availability of detailed clinical and epidemiological data, together with the relatively limited number of VP1 sequences, restricted the interpretation of the molecular and predicted epitope findings in relation to the current molecular epidemiology, genetic diversity, and epidemiological characteristics of BKoV in Türkiye.

## 5. Conclusions

Calf diarrhea continues to cause significant economic losses for cattle breeders. Despite the discovery of new viruses that cause this disease, routine diagnoses are typically limited to the identification of a few known viruses. Consequently, the true role of these novel pathogens in diarrheal cases remains poorly understood, and their actual prevalence remains unknown. BKoVs, although reported in multiple countries, have not yet been comprehensively investigated. In conclusion, this study provides insight into the detection and molecular epidemiology of BKoVs in cattle in Türkiye and presents an integrated sequence- and structure-based analysis of VP1 B-cell epitopes. The combined linear and discontinuous epitope mapping approach identified B-cell epitope regions within the VP1 protein that were predicted to be structurally exposed. These findings provide preliminary targets for further experimental validation and comparative antigenic analyses across VP1 lineages to confirm the biological relevance of the predicted epitopes.

## Figures and Tables

**Table 1:** Genome organization and region lengths of BKoV strain K056/2008/TUR.

Protein Coding Region		Genome Structure	Length (nt)
		5′UTR	420
		Leader protein (L)	561
Structural Protein	P1(2571 nt)	VP0	1101
		VP3	669
		VP1	801
Non-structural Protein	P2(1902 nt)	2A	402
		2B	495
		2C	1005
	P3 (2358 nt)	3A	382
		3B	90
		3C	576
		3D	1410
		3′UTR	138

**Table 2:** Conserved and partially conserved linear B-cell epitope regions of the VP1 protein were identified across BKoVs based on overlapping predictions of BepiPred 2.0 and Kolaskar & Tongaonkar antigenicity methods. The table was organized to facilitate comparison of shared, partially overlapping, and fragmented predicted epitope regions among strains.

Strain	Epitope ID	Start	End	Length (aa)	Epitope
K056/2008/TUR	K056-1	64	73	10	AAGVHSVTYN
	K056-2	140	149	10	LANCFTVDAQ
	K056-3	248	256	9	IVKLPVYRP
URFA2/2014/TUR	URFA2-1	64	73	10	AAGVHSVTYN
	URFA2-2	149	149	1	Q
	URFA2-3	249	256	8	VKLPVYRP
4950/2015/TUR	4950-1	64	73	10	TAGVHSVTYN
	4950-2	140	141	2	LA
	4950-3	145	149	5	TVDAQ
	4950-4	248	257	10	IVKLPVYRPM
DG10/2020/TUR	DG10-1	67	73	7	IHSVTYN
	DG10-2	149	149	1	Q
	DG10-3	248	256	9	IVKLPVYRP
DG13/2020/TUR	DG13-1	67	72	6	IHSVTY
	DG13-2	140	149	10	LANCFTVDAQ
	DG13-3	248	256	9	IVKLPVYRP
DG15/2020/TUR	DG15-1	67	73	7	IHSVTYN
	DG15-2	145	149	5	TVDAQ
	DG15-3	248	256	9	IVKLPVYRP
DG21/2020/TUR	DG21-1	64	73	10	TAGVHSVTYN
	DG21-2	140	140	1	L
	DG21-3	145	145	1	T
	DG21-4	147	149	3	DAQ
	DG21-5	248	256	9	IVKLPVYRP

## Data Availability

Data available in a publicly accessible repository: The data presented in this study are openly available in the GenBank database under the accession numbers PZ284860-PZ284866.

## Supplementary Materials

The following supporting information can be downloaded at: https://www.mdpi.com/article/10.3390/ani16131973/s1. Table S1: List of primers used in this study; Table S2: Nucleotide sequence identities (%) between the BKoV strain K056/2008/TUR and the reference strains across different genomic regions; Table S3: Amino acid sequence identities (%) between the BKoV strain K056/2008/TUR and the reference strains across different genomic regions; Table S4: Sequence-based linear B-cell epitope predictions in the VP1 protein of seven BKoV strains using BepiPred 2.0 and the Kolaskar & Tongaonkar antigenicity methods. Epitopes commonly predicted by both methods are shown in bold; Table S5: Complete list of ElliPro-predicted discontinuous B-cell epitopes of VP1 proteins from seven BKoV strains, including residue composition, epitope length, and ElliPro scores. Table S6: Co-infections detected in BKoV-positive animals and sampled farms; Figure S1: SimPlot analysis of K056/2008/TUR. Similarity plot analysis using K056/2008/TUR as the query showed variable similarity to OP805597.1 and ON168738.1 across the genome. SimPlot analysis was performed using a window size of 200 nt and a step size of 20 nt.
