# Supplementary material for: Detection and Genetic Diversity of Bovine Kobuvirus in Diarrheic Calves in Türkiye: Genomic Characterization and Comparative B-Cell Epitope Analysis
Source: Animals (Basel). 2026 Jun 26;16(13):1973. doi: 10.3390/ani16131973 (PMC13359862; doi:10.3390/ani16131973)
Supplement: Supplementary file 1 [file animals-16-01973-s001.zip › animals-4343817-supplementary.pdf]

Supplementary Materials - Detection and Genetic Diversity of Bovine Kobuvirus ins Diarrheic Calves in Türkiye: Genomic Characterization and Comparative B-Cell Epitope Analysis

Selda Duran-Yelken [1,†], İlke Karayel-Hacıoğlu [2,3,†], Krisztián Bányai [4,5,6], Szilvia Marton [7,8] and Feray Alkan [2,3,*]

[1] Department of Virology, Faculty of Veterinary Medicine, Kastamonu University, Kastamonu 37150, Türkiye; syelken@kastamonu.edu.tr (S.D.Y.)
[2] Department of Virology, Faculty of Veterinary Medicine, Ankara University, Ankara 06070, Türkiye; ikarayel@ankara.edu.tr (İ.K.H); falkan@ankara.edu.tr (F.A.)
[3] Department of Virology, Graduate School of Health Sciences, Ankara University, Ankara 06070, Türkiye
[4] Department of Medical Biology, Medical School, University of Pécs, 7624 Pécs, Hungary; bkrota@hotmail.com (B.K.)
[5] Department of Pharmacology and Toxicology, University of Veterinary Medicine, 1078 Budapest, Hungary
[6] HUN-REN Veterinary Medical Research Institute, 1143 Budapest, Hungary
[7] HUN-REN-PE Limnoecology Research Group, 8200 Veszprém, Hungary; martonsil@gmail.com (S.M.)
[8] Research Group of Limnology, Center of Natural Sciences, University of Pannonia, 8200 Veszprém,Hungary
* Correspondence: falkan@ankara.edu.tr (F.A.) Tel.: +90-312-317-0315
† These authors contributed equally to this work.

Table S1. List of primers used in this study

Primer name	Sequence (5′-3′)	Size (bp)	References
**BKoV-3D-F**	GTC TCG AGC CTG GAA CCA AA	1032	[1]
**BKoV-3D-R**	GAC GCT GGT CAT CTG GAA CA		
**BKoV-VP1-F**	TCC AAC TCC TTY TCT GTY CGG	890	[1]
**BKoV-VP1-R**	GAG CAG AGA CAG GGT TGG AA		
**BKoV-2BC-F**	ACC GTG TCT CAA GAT CCA TT	844	[1]
**BKoV-2BC-R**	GGC CCA TAC AGG TAA ATC AC		
**BKoV-3-F**	CAA CAT TTA CGG YAA CGG GAA CAA CG	690	[2]
**BKoV-3-R**	CAR TCY GGR TAC GCY TGC ACR AA		
**BKoV-7-F**	TCT TGC CGA CTC CCA YGA TGA AA	1128	[2]
**BKoV-7-R**	ATC GGR ACR CCT TGG AAA GCA GA		
**BKoV-8-F**	TCT TCG CYA CBA CCA CCT TYC TC	853	[2]
**BKoV-8-R**	CGG CCT GRT TCA TGT CCA YDC CA		

References

1. Mohamed, F.F.; Mansour, S.M.G.; Orabi, A.; El-Araby, I.E.; Ng, T.F.F.; Mor, S.K.; Goyal, S.M. Detection and Genetic Characterization of Bovine Kobuvirus from Calves in Egypt. *Arch. Virol.* **2018**, *163*, 1439–1447, doi:10.1007/s00705-018-3758-1.
2. Li, H.; Tang, C.; Yue, H. Molecular Detection and Genomic Characteristics of Bovine Kobuvirus from Dairy Calves in China. *Infection, Genetics and Evolution* **2019**, *74*, 103939, doi:10.1016/j.meegid.2019.103939.

Table S2. Nucleotide sequence identities (%) between the BKoV strain K056/2008/TUR and the reference strains across different genomic regions.

	L	VP0	VP3	VP1	2A	2B	2C	3A	3B	3C	3D	P1	P2	P3	CDS
**BKV**	100	100	100	100	100	100	100	100	100	100	100	100	100	100	100
**ON075050**	89.52	84.78	82.21	85.82	90.29	91.91	93.73	95.57	95.55	94.61	92.97	84.43	92.53	93.79	89.89
**ON075051**	88.13	84.51	82.36	85.57	91.04	93.93	92.13	94.89	96.66	93.75	91.63	84.28	92.37	92.74	89.35
**ON075052**	88.83	85.41	81.46	82.71	88.8	92.72	92.53	95.23	97.77	94.44	93.47	83.54	91.79	94.09	89.43
**ON075053**	88.83	86.5	83.4	85.57	93.03	92.72	93.53	93.87	95.55	93.92	93.19	85.4	93.21	93.54	90.27
**ON075054**	86.73	85.41	82.36	85.07	90.04	92.32	91.54	93.53	96.66	94.27	92.12	84.51	91.43	92.99	89.16
**ON075055**	87.95	83.6	81.01	85.57	88.55	93.33	90.74	91.49	95.55	93.57	92.69	83.54	90.95	92.86	88.76
**ON075056**	89.35	84.96	82.81	85.19	89.8	90.9	91.44	95.57	96.66	94.96	92.05	84.47	90.95	93.37	89.35
**MK080265**	88.65	84.78	82.81	85.94	89.8	92.72	92.33	95.91	96.66	95.48	92.83	84.63	91.9	94	89.8
**KY407744**	83.42	89.13	84.75	87.18	89.8	88.48	90.64	91.49	95.55	93.4	93.82	87.38	89.9	93.5	89.67
**MN336260**	81.84	84.69	86.39	86.44	91.04	91.91	90.74	91.15	97.77	91.84	95.95	85.68	91.11	94.43	89.57
**MW605074**	84.64	84.87	86.99	86.06	88.8	88.88	90.74	91.49	96.66	92.18	94.68	85.79	89.85	93.75	89.28
**AB084788**	81.84	86.68	86.39	86.94	88.8	85.85	89.15	89.79	95.55	92.18	92.62	86.68	88.22	92.27	88.49
**KT003671**	81.32	86.77	85.65	87.06	89.55	87.87	89.85	88.09	96.66	89.4	93.4	86.57	89.27	91.89	88.56
**MZ603734**	89.17	85.05	81.61	85.32	92.53	91.51	92.23	94.55	98.88	94.79	93.54	84.24	92.11	94.17	89.81
**ON168737**	82.37	84.87	82.06	82.46	89.3	91.71	91.14	94.21	96.66	95.65	93.47	83.39	90.9	94.21	88.69
**ON168738**	83.24	86.41	81.91	81.46	90.54	92.12	92.83	95.23	97.77	95.13	92.9	83.7	92.16	93.92	89.09
**ON168739**	89.52	86.05	82.95	83.2	90.29	91.71	93.03	94.89	97.77	94.09	93.19	84.36	92.11	93.79	89.76
**ON168740**	84.46	78.53	78.62	75.37	90.54	92.52	92.63	95.23	97.77	95.13	93.33	77.57	92.16	94.17	87.14
**ON168741**	89.35	84.51	82.81	86.44	90.29	93.13	91.64	94.21	95.55	93.22	93.04	84.67	91.74	93.33	89.61
**ON168742**	89	85.5	81.76	85.44	89.55	93.13	91.24	95.23	98.88	93.92	92.34	84.51	91.37	93.33	89.43
**OP805596**	87.26	88.85	86.69	88.3	88.3	88.28	89.25	90.47	94.44	90.45	93.82	88.12	88.8	92.61	89.66
**OP805597**	86.03	88.76	86.84	88.18	87.81	88.08	88.95	89.79	93.33	90.1	93.61	88.08	88.48	92.27	89.36
**OP805598**	85.16	88.94	86.54	88.18	88.55	87.87	88.95	90.47	94.44	90.45	93.54	88.08	88.59	92.44	89.38
**OK019998**	84.81	82.6	78.92	75.12	89.8	90.7	92.43	94.55	96.66	93.75	92.34	79.31	91.43	93.12	87.25
**PV410179**	89	85.68	82.66	85.69	90.54	93.93	92.93	93.53	97.77	95.65	93.04	84.9	92.69	93.92	90.09
**PV410180**	88.83	85.86	82.95	86.06	90.79	92.12	93.33	94.21	97.77	95.13	93.75	85.17	92.48	94.3	90.24
**PX132362**	88.3	85.96	82.51	85.69	90.54	91.51	92.63	96.93	96.66	95.31	93.12	84.98	91.9	94.26	89.97
**PQ360973**	89.35	88.04	85.2	86.56	88.55	88.88	91.24	92.85	96.66	91.31	94.25	86.84	90.06	93.45	89.97
**PV797393**	87.43	84.32	82.36	84.95	90.54	93.33	92.93	95.57	97.77	93.92	93.4	84.01	92.53	93.96	89.63
**PQ583799**	83.24	78.35	79.82	75.12	90.54	89.69	90.94	92.17	96.66	91.31	95.1	77.72	90.53	93.88	86.59
**OP920760**	89	84.78	82.06	85.19	90.79	92.72	93.23	95.57	98.88	93.75	91.91	84.2	92.58	93.08	89.55
**GU245693**	64.74	77.53	78.62	75.24	78.1	84.24	85.87	80.95	85.55	86.45	90.7	77.1	83.8	88.27	81.43

Table S3. Amino acid sequence identities (%) between the BKoV strain K056/2008/TUR and the reference strains across different genomic regions.

	L	VP0	VP3	VP1	2A	2B	2C	3A	3B	3C	3D	P1	P2	P3	CDS
**K056/2008/TUR**	100	100	100	100	100	100	100	100	100	100	100	100	100	100	100
**ON075050**	94.24	93.47	94.61	94.4	97.76	100	99.4	100	96.66	98.43	99.14	94.06	99.21	98.98	96.96
**ON075051**	91.62	93.75	94.61	94.02	97.76	99.39	100	97.95	100	98.95	98.51	94.06	99.36	98.6	96.68
**ON075052**	91.62	93.75	95.51	91.79	98.5	100	99.7	98.97	100	97.91	98.72	93.59	99.52	98.6	96.56
**ON075053**	95.28	94.56	96.41	94.77	98.5	98.78	99.1	98.97	100	98.43	98.51	95.11	98.89	98.6	97.21
**ON075054**	91.62	94.02	92.82	92.53	97.76	99.39	99.1	98.97	100	98.43	98.08	93.24	98.89	98.35	96.2
**ON075055**	92.14	93.47	95.96	93.65	96.26	98.78	99.4	98.97	96.66	98.43	98.72	94.17	98.58	98.6	96.56
**ON075056**	94.24	93.75	94.17	93.28	97.01	96.96	99.1	100	100	98.43	98.08	93.71	98.1	98.48	96.4
**MK080265**	94.24	92.66	95.51	94.02	97.76	98.78	99.4	98.97	100	98.95	98.72	93.83	98.89	98.86	96.76
**KY407744**	89.52	96.73	96.86	94.4	96.26	94.54	98.2	97.95	100	97.91	97.87	96.04	96.84	97.97	96.36
**MN336260**	83.76	94.83	97.75	92.91	98.5	95.15	97.91	93.87	100	95.31	98.29	94.99	97.31	97.08	95.39
**MW605074**	87.43	95.1	97.75	92.91	96.26	93.93	96.41	97.95	96.66	96.35	98.51	95.11	95.74	97.84	95.55
**AB084788**	83.76	93.47	95.51	92.91	96.26	87.87	95.82	95.91	96.66	96.35	98.08	93.83	93.84	97.34	94.17
**KT003671**	84.29	96.73	96.86	94.4	97.01	94.54	97.61	94.89	96.66	95.83	97.65	96.04	96.68	96.83	95.55
**MZ603734**	94.24	93.47	95.96	94.4	97.76	100	99.4	98.97	96.66	97.91	98.72	94.41	99.21	98.48	96.92
**ON168737**	87.95	94.83	95.06	92.16	97.01	100	99.4	100	96.66	98.95	98.08	94.06	99.05	98.48	96.28
**ON168738**	87.95	93.2	95.51	91.41	97.01	99.39	100	100	100	98.95	98.72	93.24	99.21	98.98	96.2
**ON168739**	94.76	95.38	95.51	93.28	97.01	100	100	98.97	100	98.95	98.72	94.76	99.36	98.86	97.25
**ON168740**	90.05	85.86	85.65	83.95	97.76	97.57	99.7	100	100	98.95	98.72	85.21	98.73	98.98	93.45
**ON168741**	93.71	93.47	95.51	95.52	97.76	98.78	100	100	96.66	98.95	98.93	94.64	99.21	98.98	97.13
**ON168742**	93.71	93.47	94.17	94.02	97.76	100	99.4	100	100	98.43	98.51	93.83	99.21	98.73	96.76
**OP805596**	90.57	96.73	97.75	95.14	97.01	93.93	96.71	95.91	96.66	96.35	98.29	96.5	96.05	97.46	96.24
**OP805597**	89.52	96.73	97.75	95.14	96.26	93.93	96.41	95.91	96.66	96.35	98.29	96.5	95.74	97.46	96.07
**OP805598**	88.48	97.01	97.75	94.77	97.01	93.93	96.41	95.91	96.66	96.35	98.29	96.5	95.89	97.46	96.03
**OK019998**	91.09	88.04	88.78	82.83	97.76	96.96	99.4	96.93	96.66	98.43	98.08	86.61	98.42	97.97	93.61
**PV410179**	94.24	93.75	94.61	94.4	97.76	99.39	99.4	100	100	98.95	98.93	94.17	99.05	99.11	97
**PV410180**	93.71	94.56	94.61	93.65	96.26	98.78	99.4	100	100	98.43	99.14	94.29	98.58	99.11	96.88
**PX132362**	93.19	93.47	95.06	93.65	98.5	99.39	99.7	100	100	98.43	98.51	93.94	99.36	98.73	96.8
**PQ360973**	93.71	97.82	98.2	92.16	97.01	95.15	97.61	94.89	100	96.87	98.72	96.15	96.84	97.84	96.68
**PV797393**	91.62	93.47	95.51	93.65	97.76	99.39	99.4	100	100	97.91	98.93	94.06	99.05	98.86	96.68
**PQ583799**	87.43	83.96	87.89	78.73	97.01	95.15	97.91	96.93	100	95.83	98.72	83.35	97	97.84	91.79
**OP920760**	94.24	93.2	94.61	93.28	97.01	99.39	99.7	100	96.66	98.95	97.65	93.59	99.05	98.22	96.52
**GU245693**	59.68	84.51	84.3	80.97	85.07	87.87	93.13	86.73	90	88.54	95.74	83.35	90.06	92.65	86.21

Table S4. Sequence-based linear B-cell epitope predictions in the VP1 protein of seven BKoV strains using BepiPred 2.0 and the Kolaskar & Tongaonkar antigenicity methods. Epitopes commonly predicted by both methods are shown in **bold**.

**Strain**	**Kolaskar & Tongaonkar**			**BepiPred 2.0**		
	**Start**	**End**	**Epitops**	**Start**	**End**	**Epitops**
**K056/2008/TUR**	45	52	YFSVYRPV	5	6	IE
	64	73	**AAGVHSVTYN**	8	41	PTPVSNLENGEATHTPEPRTTFQYTDNPSTPETN
	84	94	GDTLPLLLSAF	56	82	GNQYGISA**AAGVHSVTYN**PVEWIANAQ
	113	124	FTAQVTFHYFPP	125	155	GATPYDASSPNASTD**LANCFTVDAQ**IPPTSM
	140	149	**LANCFTVDAQ**	178	187	FSNFQGGNDV
	156	174	DTVCLSFPYLSVLSAIPTS	204	208	DVPAG
	193	200	GTIVIYVG	232	263	KLTAYTQPSTTTKTNA**IVKLPVYRP**MATTQAM
	210	216	ALTIFQK			
	248	256	**IVKLPVYRP**			
**URFA2/2014/TUR**	45	52	YFSVYRPV	6	42	EAPTPISNLENGEATHTPEPRTTFQFSDNPSTPETNL
	64	73	**AAGVHSVTYN**	59	82	YGITA**AAGVHSVTYN**PVEWIANAQ
	84	96	GDTLPLLLSCFTY	125	138	GATPYDATSPNAST
	113	123	YTAQVTFHYFP	149	155	**Q**IPPTSM
	140	149	LANCFTVDA**Q**	178	188	FSNFQGGSDIL
	156	174	DTVCLSFPYLSVLSAIPTS	204	208	DVPSG
	193	200	GTIVIYVG	232	245	KLTAYSQPNMTTKT
	211	217	LTIYQKI	249	263	**VKLPVYRP**MAAAKAM
	246	256	NAV**VKLPVYRP**			
**4950/2015/TUR**	45	52	YFSVYRPV	14	43	LENGEATHSPEPRTTFQYTDNPSTPETNLR
	64	73	**TAGVHSVTYN**	58	74	QYGITA**TAGVHSVTYN**P
	84	94	GDTLPLLLSAF	82	82	Q
	113	123	YTAQVTFHYFP	125	141	GATPYDASSPNASTDLA

	140	149	LANCF**TVDAQ**	145	155	**TVDAQ**IPPTSM
	156	174	DTVCLSFPYLSVLSAIPTS	179	188	SNFQGGSDVL
	193	200	GTIVIYVG	205	208	VPAG
	211	217	LTIYQKI	234	262	TAYSQPDTTTKTNA**IVKLPVYRP**MAVAKA
	248	257	**IVKLPVYRPM**			
**DG10/2020/TUR**	45	52	YFSVYRPV	5	6	IE
	67	73	**IHSVTYN**	8	41	PTPVSNLENGEATHTPEPRTTFQYTDNPSTPETN
	84	95	GDTLPLLLSAFT	56	81	GNNYGITATAG**IHSVTYN**PVEWIANA
	113	124	FTAQVTFHYFPP	125	139	GATPYDASSPNASTD
	140	149	LANCFTVDA**Q**	149	155	**Q**IPPTSM
	156	175	DTVCLSFPYLSVLSAIPTSY	178	188	FSNFQGGSDVL
	193	200	GTIVIYVG	205	208	VPTG
	211	217	LTIFQKI	231	261	PKLTAYTQPNMTTKTNA**IVKLPVYRP**MAAAK
	248	256	**IVKLPVYRP**			
**DG13/2020/TUR**	45	52	YFSVYRPV	5	41	IEAPTPVSNLENGEATHTPEPRTTFQYTDNPSTPETN
	67	73	**IHSVTY**N	59	72	YGIAATAG**IHSVTY**
	84	94	GDTLPLLLSAF	125	155	GATPYDASSPNASTD**LANCFTVDAQ**IPPTSM
	113	124	FTAQVTFHYFPP	179	188	SNFQGGSDVL
	140	149	**LANCFTVDAQ**	206	208	PTG
	156	174	DTVCLSFPYLSVLSAIPTS	232	262	KLTAYTQPNMTTKTNA**IVKLPVYRP**MAAAKA
	193	200	GTIVIYVG			
	211	217	LTIFQKI			
	248	256	**IVKLPVYRP**			
**DG15/2020/TUR**	45	52	YFSVYRPV	5	6	IE
	67	73	**IHSVTYN**	8	43	PTPVSNLENGEATHTPEPRTTFQYTDNPSNPETNQR

	84	95	GDTLPLLLSAFT	64	74	TAG**IHSVTYNP**
	113	124	FTAQVTFHYFPP	125	141	GATPYDASSPNASTDLA
	140	149	LANCF**TVDAQ**	145	154	**TVDAQ**IPPTS
	156	175	DTVCLSFPYLSVLSAIPTSY	178	188	FSNFQGGSDVL
	193	200	GTIVIYVG	205	208	VPTG
	211	217	LTIFQKI	232	259	KLTAYTQPNMTTKTNA**IVKLPVYRP**MAA
	248	256	**IVKLPVYRP**	261	261	K
**DG21/2020/TUR**	45	52	YFSVYRPV	5	6	IE
	64	73	**TAGVHSVTYN**	9	40	TPVSNLENGEATHTPEPRTTFQYTDNPTPPET
	84	94	GDTLPLLLSAF	61	73	ITA**TAGVHSVTYN**
	113	123	YTAQVTFHYFP	77	82	WIANAQ
	140	149	**L**ANCF**T**V**DAQ**	125	140	GATPYDASSPNASTD**L**
	156	174	DTVCLSFPYLSVLSAIPTS	145	145	**T**
	193	200	GTIVIYVG	147	155	**DAQ**IPPTSM
	211	217	LTIYQKI	178	188	FSNFQGGSDVL
	248	256	**IVKLPVYRP**	204	208	DVPAG
				233	261	LTAYSQPDMTTRTNA**IVKLPVYRP**MAATK

Table S5. Complete list of ElliPro-predicted discontinuous B-cell epitopes of VP1 proteins from seven BKoV strains, including residue composition, epitope length, and ElliPro scores.

Strain	No	Residues	Length (aa)	Score
**K056/2008/TUR**	1	A:A1, A:G2, A:D3, A:G4, A:I5, A:E6, A:A7, A:P8, A:T9, A:P10, A:V11, A:S12, A:N13, A:L14, A:E15, A:N16, A:G17, A:E18, A:A19, A:T20, A:H21, A:T22, A:P23, A:E24, A:P25	25	0.901
	2	A:L233, A:T234, A:A235, A:Y236, A:T237, A:Q238, A:P239, A:S240, A:T241, A:T242, A:T243, A:K244, A:T245, A:N246, A:A247, A:I248, A:V249, A:K250, A:L251, A:P252, A:V253, A:R255, A:P256, A:M257, A:A258, A:T259, A:T260, A:Q261, A:A262, A:M263, A:V264	31	0.855
	3	A:R26, A:T27, A:T28, A:F29	4	0.755
	4	A:A64, A:A65, A:G66, A:S108, A:N109, A:P110, A:A111, A:A112, A:F113, A:T114, A:A115, A:Q116, A:D147, A:A148, A:Q149, A:I150, A:P151, A:P152, A:T153, A:S154, A:M155, A:D156, A:Y198, A:V199, A:G200, A:W201, A:Q202, A:G203, A:D204, A:V205, A:P206, A:A207, A:G208, A:T209, A:A210, A:L211	36	0.643
	5	A:Y31, A:T32, A:D33, A:N34, A:P35, A:S36, A:T37, A:P38	8	0.632
	6	A:Y129, A:D130, A:A131, A:S132, A:S133, A:P134, A:N135, A:A136, A:S137, A:T138, A:D139, A:L140, A:A141, A:N142, A:C143	15	0.588
	7	A:N229, A:P230, A:P231, A:K232	4	0.574
	8	A:S179, A:N180, A:F181, A:Q182, A:G183, A:G184, A:N185	7	0.563
**URFA2/2014/TUR**	1	A:L233, A:T234, A:A235, A:Y236, A:S237, A:Q238, A:P239, A:N240, A:M241, A:T242, A:T243, A:K244	12	0.917
	2	A:L14, A:E15, A:N16, A:G17, A:E18, A:A19, A:T20, A:H21, A:T22, A:P23, A:E24, A:P25	12	0.91
	3	A:G2, A:D3, A:G4, A:I5, A:E6, A:A7, A:P8, A:T9, A:P10, A:I11, A:S12, A:N13	12	0.905
	4	A:T27, A:T28, A:F29	3	0.852
	5	A:T245, A:N246, A:A247, A:V248, A:V249, A:K250, A:L251, A:P252, A:V253, A:Y254, A:R255, A:P256, A:M257, A:A258, A:A259, A:A260, A:K261, A:A262, A:M263, A:V264, A:R265, A:R266, A:Q267	23	0.699
	6	A:A64, A:A65, A:S108, A:N109, A:P110, A:A111, A:A112, A:Y113, A:T114, A:A115, A:Q116, A:Q149, A:I150, A:P151, A:P152, A:T153, A:S154, A:Y198, A:V199, A:G200, A:W201, A:Q202, A:G203, A:D204, A:V205, A:P206, A:S207, A:G208, A:T209, A:A210	30	0.691
	7	A:N229, A:P230, A:P231, A:K232	4	0.662
	8	A:Y129, A:D130, A:A131, A:T132, A:S133, A:P134, A:N135, A:A136, A:S137, A:T138, A:D139, A:L140, A:A141, A:N142, A:C143	15	0.639
	9	A:G177, A:F178, A:S179, A:N180, A:F181, A:Q182, A:G183, A:G184, A:S185, A:D186, A:I187, A:L188, A:N189	13	0.6
	10	A:F31, A:S32, A:D33, A:N34, A:P35, A:S36	6	0.558
**4950/2015/TUR**	1	A:P10, A:V11, A:S12, A:N13, A:L14, A:E15, A:N16, A:G17, A:E18, A:A19, A:T20, A:H21, A:S22, A:P23, A:E24, A:P25	16	0.936
	2	A:T245, A:N246, A:A247, A:I248, A:V249, A:K250, A:L251, A:P252, A:V253, A:Y254, A:R255, A:P256, A:M257, A:A258, A:V259, A:A260, A:K261, A:A262, A:M263, A:V264	20	0.859
	3	A:R26, A:T27, A:T28, A:F29	4	0.828

	4	A:R228, A:N229, A:P230, A:P231, A:K232, A:L233, A:T234, A:A235, A:Y236, A:S237, A:Q238, A:P239, A:D240, A:T241, A:T242, A:T243, A:K244	17	0.783
	5	A:Y31, A:T32, A:D33, A:N34, A:P35, A:S36, A:T37, A:P38, A:E39	9	0.671
	6	A:T64, A:A65, A:G66, A:V67, A:N109, A:P110, A:A111, A:A112, A:Y113, A:T114, A:A115, A:Q116, A:D147, A:A148, A:Q149, A:I150, A:P151, A:P152, A:T153, A:S154, A:M155, A:D156, A:Y198, A:V199, A:G200, A:W201, A:Q202, A:G203, A:D204, A:V205, A:P206, A:A207, A:G208, A:T209, A:A210	35	0.648
	7	A:Y129, A:D130, A:A131, A:S132, A:S133, A:P134, A:N135, A:A136, A:S137, A:T138, A:D139, A:L140, A:A141, A:N142, A:C143	15	0.64
	8	A:G183, A:G184, A:S185, A:D186	4	0.525
	9	A:F178, A:S179, A:N180, A:F181	4	0.509
**DG10/2020/TUR**	1	A:L14, A:E15, A:N16, A:G17, A:E18, A:A19, A:T20, A:H21, A:T22, A:P23, A:E24, A:P25, A:T27, A:T28, A:F29, A:Y31	16	0.891
	2	A:L233, A:T234, A:A235, A:Y236, A:T237, A:Q238, A:P239, A:N240, A:M241, A:T242, A:T243, A:K244, A:T245, A:N246, A:A247, A:I248, A:V249, A:K250	18	0.873
	3	A:A1, A:G2, A:D3, A:G4, A:I5, A:E6, A:A7, A:P8, A:T9, A:P10, A:V11, A:S12, A:N13	13	0.84
	4	A:L251, A:P252, A:V253, A:R255, A:P256, A:M257, A:A258, A:A259, A:A260, A:K261, A:A262, A:M263, A:V264	13	0.836
	5	A:T32, A:D33, A:N34, A:P35, A:S36, A:T37, A:P38	7	0.635
	6	A:T62, A:T64, A:A65, A:G66, A:S108, A:N109, A:P110, A:A111, A:A112, A:F113, A:T114, A:A115, A:Q116, A:D147, A:A148, A:Q149, A:I150, A:P151, A:P152, A:T153, A:S154, A:M155, A:D156, A:Y198, A:V199, A:G200, A:W201, A:Q202, A:G203, A:D204, A:V205, A:P206, A:T207, A:G208, A:T209, A:A210, A:L211	37	0.626
	7	A:Y129, A:D130, A:A131, A:S132, A:S133, A:P134, A:N135, A:A136, A:S137, A:T138, A:D139, A:A141, A:N142, A:C143	14	0.602
	8	A:Y175, A:G177, A:F178, A:S179, A:N180, A:F181, A:Q182, A:G183, A:G184, A:S185	10	0.598
	9	A:N229, A:P230, A:P231, A:K232	4	0.549
**DG13/2020/TUR**	1	A:L14, A:E15, A:N16, A:G17, A:E18, A:A19, A:T20, A:H21, A:T22, A:P23, A:E24, A:P25	12	0.933
	2	A:L251, A:P252, A:V253, A:R255, A:P256, A:M257, A:A258, A:A259, A:A260, A:K261, A:A262, A:M263, A:V264	13	0.883
	3	A:A1, A:G2, A:D3, A:G4, A:I5, A:E6, A:A7, A:P8, A:T9, A:P10, A:V11, A:S12, A:N13	13	0.862
	4	A:L233, A:T234, A:A235, A:Y236, A:T237, A:Q238, A:P239, A:N240, A:M241, A:T242, A:T243, A:K244, A:T245, A:N246, A:A247, A:I248, A:V249, A:K250	18	0.818
	5	A:T27, A:T28, A:F29	3	0.748
	6	A:A62, A:T64, A:A65, A:S108, A:N109, A:P110, A:A111, A:A112, A:F113, A:T114, A:A115, A:Q116, A:D147, A:A148, A:Q149, A:I150, A:P151, A:P152, A:T153, A:S154, A:M155, A:D156, A:G200, A:W201, A:Q202, A:G203, A:D204, A:V205, A:P206, A:T207, A:G208, A:T209, A:A210, A:L211, A:T212	35	0.652
	7	A:Q30, A:T32, A:D33, A:N34, A:P35, A:S36, A:T37, A:P38, A:E39, A:N41, A:R44	11	0.606
	8	A:Y129, A:D130, A:A131, A:S132, A:S133, A:P134, A:N135, A:A136, A:S137, A:T138, A:D139, A:A141, A:N142, A:C143	14	0.584
	9	A:G177, A:F178, A:S179, A:N180, A:F181, A:Q182, A:G183, A:G184, A:S185	9	0.563
**DG15/2020/TUR**	1	A:E6, A:A7, A:P8, A:T9, A:P10, A:V11, A:S12, A:N13, A:L14, A:E15, A:N16, A:G17, A:E18, A:A19, A:T20, A:H21, A:T22, A:P23, A:E24, A:P25	20	0.895

	2	A:L233, A:T234, A:A235, A:Y236, A:T237, A:Q238, A:P239, A:N240, A:M241, A:T242, A:T243, A:K244, A:T245, A:N246, A:A247, A:I248, A:V249, A:K250, A:L251, A:P252, A:V253, A:Y254, A:R255, A:P256, A:M257, A:A258, A:A259, A:A260, A:K261, A:A262, A:M263, A:V264	32	0.852
	3	A:A1, A:G2, A:D3, A:G4, A:I5	5	0.796
	4	A:R26, A:T27, A:T28, A:F29	4	0.796
	5	A:T62, A:T64, A:A65, A:S108, A:N109, A:P110, A:A111, A:A112, A:F113, A:T114, A:A115, A:Q116, A:Q149, A:I150, A:P151, A:P152, A:T153, A:S154, A:M155, A:G200, A:W201, A:Q202, A:G203, A:D204, A:V205, A:P206, A:T207, A:G208, A:T209, A:A210, A:L211, A:T212	32	0.659
	6	A:R228, A:N229, A:P230, A:P231, A:K232	5	0.596
	7	A:N34, A:P35, A:S36, A:N37	4	0.576
	8	A:Y129, A:D130, A:A131, A:S132, A:S133, A:P134, A:N135, A:A136, A:S137, A:T138, A:A141, A:N142, A:C143	13	0.556
	9	A:A81, A:Q82, A:V83, A:G84, A:D85	5	0.539
	10	A:G177, A:F178, A:S179, A:N180, A:Q182, A:G183, A:G184	7	0.529
**DG21/2020/TUR**	1	A:A1, A:G2, A:D3, A:G4, A:I5, A:E6, A:A7, A:P8, A:T9, A:P10, A:V11, A:S12, A:N13, A:L14, A:E15, A:N16, A:G17, A:E18, A:A19, A:T20, A:H21, A:T22, A:P23, A:E24, A:P25	25	0.919
	2	A:L233, A:T234, A:A235, A:Y236, A:S237, A:Q238, A:P239, A:D240, A:M241, A:T242, A:T243, A:R244, A:T245, A:N246, A:A247, A:I248, A:V249, A:K250, A:L251, A:P252, A:V253, A:Y254, A:R255, A:P256, A:M257, A:A258, A:A259, A:T260, A:K261, A:A262, A:M263, A:V264	32	0.843
	3	A:R26, A:T27, A:T28, A:F29	4	0.739
	4	A:N229, A:P230, A:P231, A:K232	4	0.652
	5	A:T62, A:A63, A:T64, A:A65, A:G66, A:V67, A:S108, A:N109, A:P110, A:A111, A:A112, A:Y113, A:T114, A:A115, A:Q116, A:D147, A:A148, A:Q149, A:I150, A:P151, A:P152, A:T153, A:S154, A:Y198, A:V199, A:G200, A:W201, A:Q202, A:G203, A:D204, A:V205, A:P206, A:A207, A:G208, A:T209, A:A210, A:L211	37	0.651
	6	A:Y175, A:G177, A:F178, A:S179, A:N180, A:F181, A:Q182, A:G183, A:G184, A:S185	10	0.585
	7	A:Y129, A:D130, A:A131, A:S132, A:S133, A:P134, A:N135, A:A136, A:S137, A:T138, A:A141, A:N142, A:C143	13	0.57
	8	A:T32, A:D33, A:N34	3	0.529

Table S6. Co-infections detected in BKoV-positive animals and sampled farms

Farm ID	City	Sampling date	Tested animals for BKoV (n)	Tested animals positive for BKoV (n)	Infection status in BKoV-positive animals		Additional infections on farm
					BKoV	Co-infection(s)	
**1**	Kırklareli	2020	4	2	DG1 and DG4	BRV (-), BCoV (-)	(-)
**2**	Kırklareli	2020	2	1	DG5	BRV (-), BCoV (-)	(-)
**3**	Konya	2020	3	2	DG10 and DG21	BRV (-), BCoV (-)	(-)
**4**	Kırklareli	2020	2	1	DG12	BRV (-), BCoV (-)	(-)
**5**	Kırklareli	2020	3	2	DG13 and DG18	BRV (-), BCoV (-)	(-)
**6**	Konya	2020	1	1	DG15	BRV (-), BCoV (-)	(-)
**7**	Kırklareli	2020	2	1	DG17	BRV (+), BCoV (-)	BRV
**8**	İzmir	2015	5	2	4949	BRV (-), BCoV (-) BNoV (-), BNeV (-)	BRV, BCoV, BNoV and BNeV

					4950	BRV (+), BCoV (+), BNoV(+), BNeV (-)	
**9**	Eskişehir	2013	2	1	460134	BRV (-), BCoV (-), BNoV (-), BNeV (+)	BRV, BNoV and BNeV
**10**	İzmir	2015	3	2	Esin and 2309008	BRV (-), BCoV (-) BNoV (-), BNeV(-)	(-)
**11**	Ankara	2015	2	1	DahA	BRV (-), BCoV (-) BNoV (+), BNeV (-)	BNoV
**12**	Bursa	2009	3	1	B3	BRV (+), BCoV (+) BNoV (-), BNeV (-)	BRV and BCoV
**13**	Bursa	2009	1	1	B18	BRV (+), BCoV (-) BNoV(+), BNeV (-)	BRV and BNoV
**14**	Şanlıurfa	2014	2	1	Urfa2	BRV (-), BCoV (-), BNoV (-),BNeV (+)	BCoV, BNoV and BNeV
**15**	İzmir	2015	3	2	9276	BRV (-), BCoV (+), BNoV(-), BNeV(+)	BRV, BCoV, and BNeV
					9283	BRV (+), BCoV (+), BNoV(-), BNeV (-)	
**16**	Aksaray	2008	1	1	K056	BRV (+), BCoV (-), BNoV(+), BNeV (+)	BRV, BCoV, BNoV and BNeV
**17**	Kastamonu	2024	18	8	A4	BRV (+), BCoV (-)	BRV and BCoV

					A2, A3, A5, A6, A8, A9, A10	BRV (-), BCoV (-)	
**18**	Ankara	2023	2	2	sec6323 and sec6325	BRV (-), BCoV (+)	BCoV
**19**	Ankara	2023	2	1	sec6643	BRV (-), BCoV (-)	BRV
**20**	Konya	2020	23	7	Kon3, Kon4, Kon6, Kon7, Kon12, Kon17 and Kon21	BRV (-), BCoV (-)	BRV

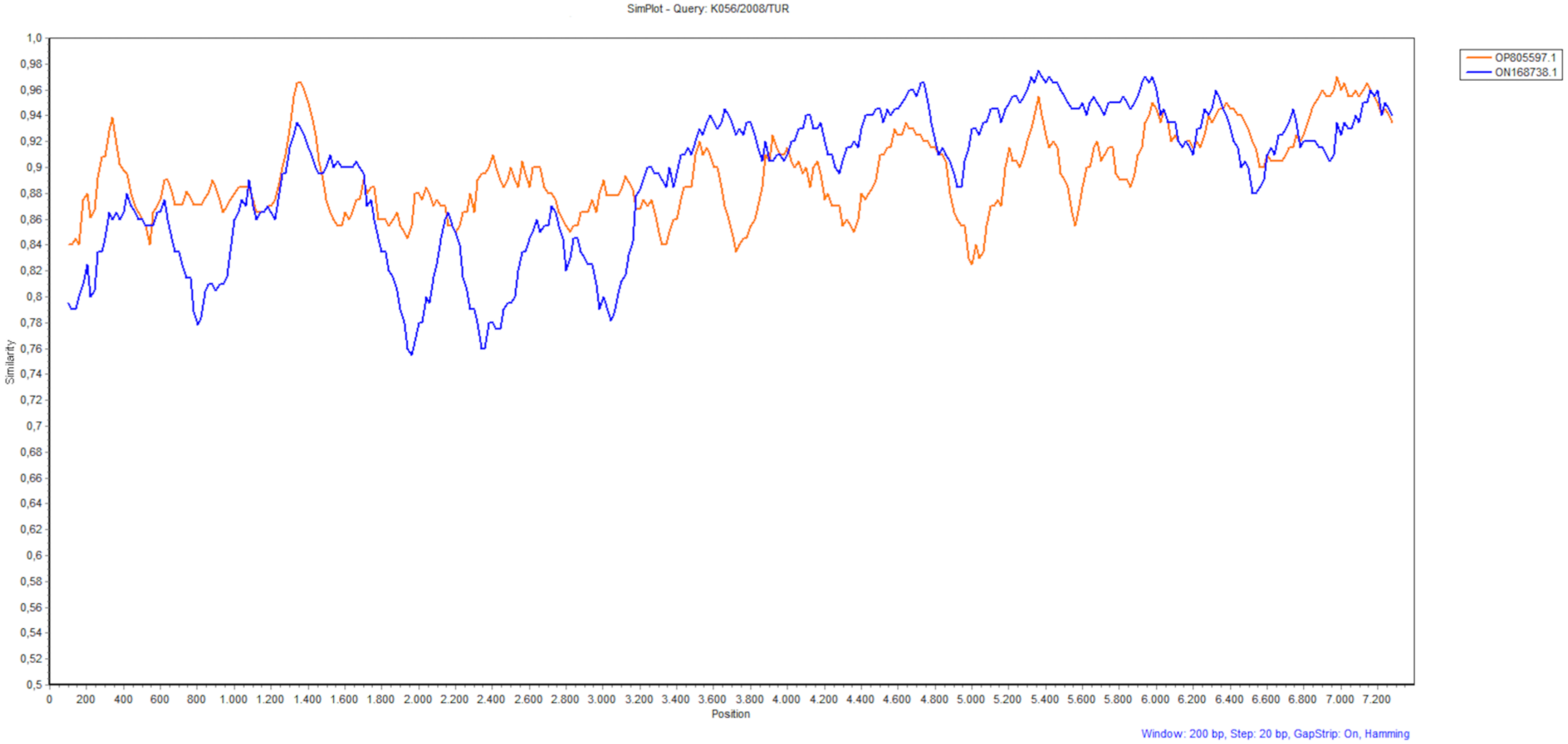

Figure S1. SimPlot analysis of K056/2008/TUR. Similarity plot analysis using K056/2008/TUR as the query showed variable similarity to OP805597.1 and ON168738.1 across the genome. SimPlot analysis was performed using a window size of 200 nt and a step size of 20 nt.
